# Supplementary material for: Asynchronous magnetic resonance elastography: Shear wave speed reconstruction using noise correlation of incoherent waves
Source: Magn Reson Med. 2022 Oct 27;89(3):990–1001. doi: 10.1002/mrm.29502 (PMC9792433; doi:10.1002/mrm.29502)
Supplement: Supplementary file 1 — DATA S1 MATLAB code used to generate the numerical simulations of Figure 1 [file MRM-89-990-s002.zip › k-Wave/helpfiles/example_ivp_binary_sensor_mask.html]

Using A Binary Sensor Mask Example (k-Wave)


# Using A Binary Sensor Mask Example

This example demonstrates how to use a binary sensor mask for the detection of the pressure field generated by an initial pressure distribution within a two-dimensional homogeneous propagation medium. It builds on the Homogeneous Propagation Medium Example.

- Open the file in the MATLAB Editor
- Run the file in MATLAB

## Contents

- Defining a binary sensor mask
- Running the simulation

## Defining a binary sensor mask

In the Homogeneous Propagation Medium Example, the sensor mask (which defines where the pressure field is recorded at each time-step) is defined as a 2 x N matrix of Cartesian points. It is also possible to define the sensor mask as binary matrix (i.e., a matrix of 1's and 0's) representing the grid points within the computational domain that will collect the data. In this case, the sensor mask must have the same dimensions as the computational grid (i.e., it must have `Nx` rows and `Ny` columns). Here `makeCircle` is used (instead of `makeCartCircle`) to define a binary sensor mask of an arc created using the midpoint circle algorithm. It is also possible to explicitly create an arbitrary binary sensor mask, or to load one from an external image map (see the Loading External Image maps Example).

```
% define a binary sensor mask
sensor_x_pos = Nx/2;        % [grid points]
sensor_y_pos = Ny/2;        % [grid points]
sensor_radius = Nx/2 - 22;  % [grid points]
sensor_arc_angle = 3*pi/2;  % [radians]
sensor.mask = makeCircle(Nx, Ny, sensor_x_pos, sensor_y_pos, sensor_radius, sensor_arc_angle);
```

A plot of the initial pressure distribution and the sensor mask is given below.


## Running the simulation

The computation is again started by calling `kspaceFirstOrder2D`. For a Cartesian sensor mask (as in the Homogeneous Propagation Medium Example), the returned time-series are ordered the same way as the set of Cartesian sensor points. For a binary sensor mask (as in this example), the time-series are instead returned using MATLAB's standard column-wise linear matrix index ordering. As an example, if the sensor mask is defined as

```
0     1     0
1     0     1
1     0     1
0     1     0
```

the ordering of the recorded time series will be

```
0     3     0
1     0     5
2     0     6
0     4     0
```

For both Cartesian and binary sensor masks, the data is indexed as `sensor_data(sensor_point_index, time_index)`. A visualisation of the recorded time data is given below. The data ordering is clearly visible; the data is returned column-wise from top to bottom, and left to right.

For 2D simulations, it is possible to reorder the sensor data returned by a binary sensor mask based on the angle that each sensor point makes with the grid origin using `reorderSensorData`. The angles are defined from the upper left quadrant or negative y-axis in the same way as within `makeCircle` and `makeCartCircle`.

```
% reorder the simulation data
sensor_data_reordered = reorderSensorData(kgrid, sensor, sensor_data);
```

The recorded data at a particular time step can also be restored to its original position in the grid using `unmaskSensorData`.
